# Cell cycle and centromere FISH studies in premature centromere division

**DOI:** 10.1186/1471-2350-6-33

**Published:** 2005-09-20

**Authors:** Alfredo Corona-Rivera, Fabio Salamanca-Gomez, Lucina Bobadilla-Morales, Jorge R Corona-Rivera, Cesar Palomino-Cueva, Teresa A Garcia-Cobian, Enrique Corona-Rivera

**Affiliations:** 1 Laboratorio de Citogenética Genotoxicidad y Biomonitoreo, Instituto de Genética Humana Dr. Enrique Corona Rivera, Departamento de Fisiología, División de Disciplinas Básicas, Centro Universitario Ciencias de la Salud, Universidad de Guadalajara, Guadalajara, Jalisco, México; 2 Unidad de Citogenética, OPD Hospital Civil Fray Antonio Alcalde, Guadalajara, Jalisco, México; 3 Unit of Investigation in Human Genetics, National Medical Center, Instituto Mexicano del Seguro Social, México City, México; 4 División de Pediatría, OPD Hospital Civil Juan I. Menchaca, Guadalajara, Jalisco, México; 5 Laboratorio de Genética Humana, Departamento de Fisiología, División de Disciplinas Básicas, Centro Universitario Ciencias de la Salud, Universidad de Guadalajara, Guadalajara, Jalisco, México

## Abstract

**Background:**

Mitotic configurations consistent in split centromeres and splayed chromatids in all or most of the chromosomes or premature centromere division (PCD) have been described in three categories. (1) Low frequency of PCD observed in colchicines-treated lymphocyte cultures from normal individuals. (2) High frequency of PCD with mosaic variegated aneuploidy. (3) High frequency of PCD as a sole chromosome abnormality observed in individuals with no recognizable clinical pattern. We report four members of a family with the third category of PCD.

**Methods:**

Cell cycle duration assessed by average generation time using differential sister chromatid stain analysis and FISH studies of DNA centromere sequences in PCD individuals, are included and compared with previously reported PCD individuals from 9 families.

**Results:**

We observed PCD in colchicine-treated cultures from the propositus, his father, and two paternal aunts but not in his mother and four other paternal and maternal family members, as well as in untreated cultures from the propositus and his father. We observed cytological evidence of active centromeres by Cd stain. Significative cell cycle time reduction in anaphases of PCD individuals (average generation time of 21.8 h;SD 0.4) with respect to individuals without PCD (average generation time of 31.8 h;SD 3.9) was observed (P < 0.005, Student t-test for independent samples). Increased cell proliferation kinetics was observed in anaphasic cells of individuals with PCD, by differential sister chromatid stain analysis. FISH studies revealed the presence of alpha satellite DNA from chromosomes 1, 13, 21/18, X, all centromeres, and CENP-B box sequences in metaphasic and anaphasic cells from PCD individuals.

**Conclusion:**

This report examines evidences of a functional relationship between PCD and cell cycle impairment. It seems that essential centromere integrity is present in these cases.

## Background

Mitotic configurations consistent in split centromeres and splayed chromatids in all or most of the chromosomes or "premature centromere division" (PCD), have been described in three categories. They are:

(1) Low frequency of PCD (up to 3% of the mitosis) observed in colchicines-treated lymphocyte cultures from normal individuals [1,2].

(2) High frequency of PCD (5% or more) with mosaic aneuploidies involving a variety of chromosomes, called "mosaic variegated aneuploidy", observed in individuals with microcephaly, growth deficiency, severe mental retardation, and risk of malignancy [3-5].

(3) High frequency of PCD (5% or more) as a sole chromosome abnormality [6-10]. Individuals with this condition have no recognizable clinical pattern or occur in healthy individuals. Association of this PCD trait with abortions and infertility has been reported [6,7,9,10], but other authors suggest this PCD trait to be harmless [1,8,11].

We report a family that corresponds to the third category of PCD, in which four individuals showed PCD as a sole chromosome abnormality. Cell cycle duration, assessed by average generation time, and FISH studies of the centromere, are considered. Findings in the family are compared with those on 30 other previously reported individuals with PCD from 9 families. This report examines the evidences of a relationship between PCD and cell cycle impairment of cells bearing main structural centromere components from PCD individuals.

## Methods

### Family data

**Figure 1:**
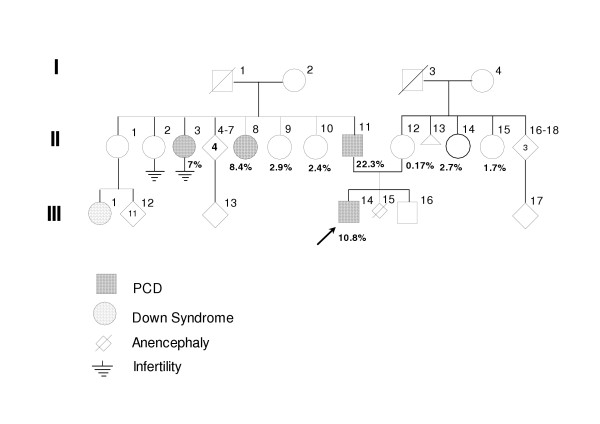
**Pedigree of the family**. Pedigree of the family. PCD frequencies and familial data of investigated individuals are indicated.

A total of nine individuals were available to be studied, five of them from the paternal lineage, four of them from the maternal lineage and the propositus. Infertility was observed in two paternal aunts. Family data are summarized on Fig. 1. The non-consanguineous parents were both aged 27 years at propositus birth. The propositus was born at 36 weeks by abdominal delivery indicated by maternal pre-eclamptic toxemia and showing neonatal hypoxia. Birth weight was 2100 g and height was 46 cm. Early signs included: hypotonia, strabismus, seizures, recurrent respiratory infections and occasional breath-holding spells. Physical examination at 5 years of age showed microcephaly (OFC -4.1 SD), low weight (-4.1 SD) and borderline length (10th percentile); plagiocephaly and brachycephaly, nystagmus, phimosis, right cryptorchidia, adducted thumbs, syndactyly 2–3 of toes, four limbs spasticity and increased deep tendon reflexes. On X-rays, hyperbrachycephaly (cephalic index 91.7), scoliosis, spina bifida occulta (T1–T5), coxa valga, slim bones and osteopenia were found. Cortical and subcortical atrophy was found on cranial CT scan. Brainstem auditory evoked potentials were normal. Psychometric test demonstrated a development age of 3–6 months. The propositus died at 10 years of age by pneumonia. Propositus relatives were phenotypically healthy.

### Cytogenetic studies

We performed GTG banded karyotypes from peripheral blood lymphocyte cultures stimulated by phytohemaglutinin and treated one hour with colchicine (SIGMA, 0.104 μg/ml), in the propositus and eight family members indicated in Fig. 1. On harvesting, 15 minutes of hypotonic treatment (0.075 M KCl at 37°C) was performed. Chromosome aberrations in the karyotypes of these individuals were not observed. PCD frequencies in repeated cultures of available individuals were obtained in at least three repeated cultures. Around 100 mitosis were scored per culture giving a total range of 258 to 1142 cells scored per individual (Table 1). To obtain basal frequencies of PCD, ten healthy young individuals were used as controls. Additional simultaneous cultures without mitostatic treatment were performed in the propositus and his parents. Cd staining was performed in the propositus and his parents according to Denton et al. (1977) [12].

### Cell cycle studies

Cellular proliferation kinetics was used to determine the cell cycle duration, which is the interval between one mitosis and the subsequent [13]. The method called average generation time (AGT) by differential sister-chromatid stain [14,15], was used to determine cell cycle durations. We compared the results between family members with more than 5% of PCD versus those with less than 3% of anaphase frequencies. The original AGT method [14,15], consider for cell cycle calculations only metaphases, in this family, calculations were also performed considering, prophases, anaphases and total mitotic cells. The procedure was as follows. We obtained AGT's of each individual from accumulated data of 3 simultaneous 72 h cultures with 5 μg/ml of 5'-bromodeoxyuridine (SIGMA), added 24 h after set-up. In each culture, we scored prophases, metaphases and anaphases which had completed either one, two or three cell cycles identified by differential sister chromatid stain pattern. Then, AGT, were calculated per mitotic stage and per individual as follows. (i) Taking into account the percentage of cells at first (M1), second (M2) and third (M3) cell cycle, the replication index (RI) was obtained from this formula: RI = (1X%M1+2X%M2+3%M3)/100. (ii) The RI was used to obtain the AGT following the equation: AGT = time of harvest after exposure to 5'-BrdU/RI. Finally, AGT's were compared in paternal individuals with PCD versus those individuals with low anaphase frequencies from the maternal sibship. Statistical test t-Student for independent samples was used to compare both groups.

### FISH studies

We searched for the presence of constitutive structural components of the centromere such as alpha satellite DNA sequences in the propositus and his parents with FISH according to a standard protocol [16], using alpha satellite DNA directly labeled probes to chromosomes 1, 13/21, 18, and X, as well as all centromeres probe. Besides, we searched for the presence of CENP-B box sequence in the propositus using a biotin labeled probe, following the protocol of Matera and Ward (1992) [17].

## Results

### Cytogenetic studies

**Figure 2:**
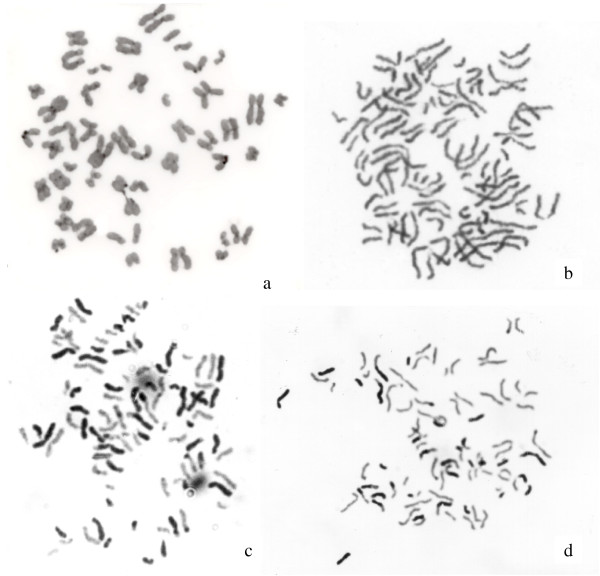
**PCD figures**. Propositus PCD figures are shown with Cd stain (a), giemsa stain (b), and sister chromatid differential stain from second (c), and third (d), cell cycle.

PCD frequencies in repeated cultures of available individuals are shown in Table 1. Individuals with PCD of 5% or more corresponded to the propositus, his father and two paternal aunts (II-3, and II-8). Individuals with less of 3% of anaphases were considered as normal and corresponded to mother's propositus, two maternal aunts (II-14, II-15), and two paternal aunts (II-9, II-10). Basal frequencies of PCD, in ten healthy young individuals used as controls, did not differ with respect to PCD individual with less of 3% of anaphases. A PCD image of the propositus is shown in figure 2a. In additional simultaneous cultures without mitostatic treatment PCD was observed in the propositus (5%) and his father (5.5%), but not in his mother. Two centromeric dots in evident primary constrictions were observed by Cd staining in the propositus and his parents (fig. 2b). This finding is considered a cytological evidence of active centromeres [12].

### Cell cycle studies

We observed cell cycle duration significatively reduced in anaphasic cells but not in prophasic, metaphasic or total cells (Table 2). Cell cycle reduction was then attributed only to cells in anaphase (PCD cells). Additionally, we observed that in the same culture conditions, 38% of anaphases in the propositus and his father reached the third cell cycle, while only 15% of metaphases did. This was interpreted as increased cell proliferation kinetics of anaphasic cells in individuals with PCD associated to cell cycle shortening in PCD cells. Anaphases from second and third cell cycle are shown in figures 2c and 2d respectively.

### FISH studies

**Figure 3:**
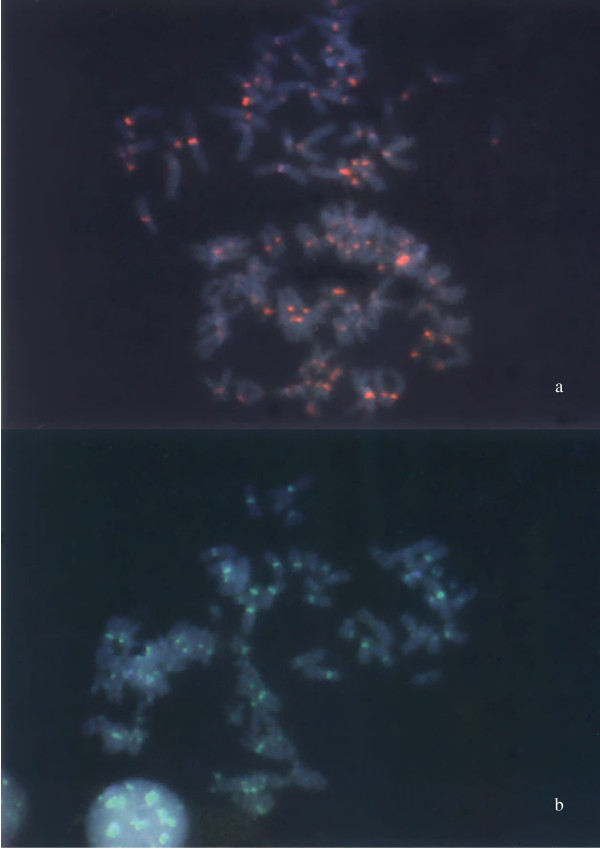
**FISH PCD images**. FISH PCD images. All centromeres FISH probe red signals (a), and CENP-B box FISH green signals (b) are shown.

We observed in all cases positive fluorescent signals (figure 3a), indicating the presence of all tested sequences. We observed a positive fluorescent signal pattern, indicating the presence of CENP-B box sequence in the propositus (figure 3b).

## Discussion

We report four family members with more than 5% of colchicine-anaphase frequencies as a sole chromosome abnormality. Nine families have been reported referring to this trait as PCD [6-10]. Mitosis obtained from colchicine arrested lymphocyte cultures of normal individuals, show rates below 3% [1], 5% [2] or 1% observed in our control individuals. Previous reports of PCD frequencies without mosaic variegated aneuploidy (MVA), ranges 5 to 38% [6-10]. In our family, PCD was observed in the propositus, his father, and two paternal aunts in repeated colchicine-treated cultures in average frequencies of 7 to 22%. It was shown [18], that PCD can be induced through hypotonic increasing time treatment of mitotic cells in peripheral blood lymphocytes of healthy individuals and patients homozygous to PCD trait or PCD and MVA. They found that 0.075 M KCl at 37°C for 20 min, showed 0–2% cells in PCD which fits with our observed frequencies because 15 minutes of hypotonic treatment were used in our peripheral blood cultures. Total mitosis scored in repeated cultures of previous [6-10], and present report did not provide evidences of MVA. Although PCD is considered a rare phenomenon, two studies found in selected population frequencies of 1 of 100 [1] or 1 of 1000 [11].

Only two previous studies tested cell cycle duration in individuals with PCD as a sole chromosome abnormality (Table 3). In both cases they obtained evidences that the cell cycle time can be altered in PCD individuals [6,7]. Rudd et al. (1983) [6], found reduced metaphase duration only in some cultured cells, supposing that such cells could have corresponded to those with PCD. Gabarrón et al. (1986) [7], inferred that only the cells in anaphase or PCD cells, showed accelerated proliferation kinetics and consequently reduced cell cycle duration. We provide evidences to confirm that cell cycle duration is reduced in PCD cells because only in anaphasic cells the cell cycle duration in the family members with PCD was statistically reduced. Additionally, we observed increased cell proliferation kinetics of anaphasic cells in individuals with PCD. These findings are compatible with the co-existence of cellular subpopulations bearing differential proliferation kinetics. Cell cycle reduction, possibly related to premature separation of centromeres and persistence of anaphases, could then be considered a distinctive finding in these cases.

Cell cycle progression requires control mechanisms that could be associated to PCD origin. A basic defect of cell cycle progression or metaphase-anaphase transition in PCD was suggested [19]. Matsuura et al. (2000) [20], demonstrated that cultured fibroblasts from two infants with PCD and mosaic variegated aneuploidy are insensitive to the colcemid-induced mitotic-spindle checkpoint. Mitchel et al. (2001) [21], found that mitotic checkpoint defective MAD2+/- haploinsufficient human colon carcinoma cells showed 20% of precocious anaphases with prematurely separated sister chromatids, compared with 1% in wild-type cells, proposing that PCD is a suitable cytogenetic marker for the identification of mitotic checkpoint defects. In our family PCD was also observed in cultures without colchicine from the propositus and his father as in other PCD reports [6-8] (Table 3). Cultured PCD cells with defective colcemid-induced mitotic-spindle checkpoint reported by Matsuura et al. (2000) [20], were unresponsive to colchicine. Although in our family MVA was not observed as in Matsuura et al. (2000) [20] report, in both cases the mitotic arrest signal was overruled in PCD cells. Hanks et al. (2004) [22], provided the evidence that gene mutations can result in a defective spindle checkpoint in humans. They screened the full coding sequence and intron-exon boundaries of *BUB1B*, and found truncating and missense mutations inherited from different parents in five of eight families with mosaic variegated aneuploidy providing the first evidence in humans that gene mutations might be responsible for aneuploidy in human cancers. Interestingly, cytogenetic data of families 1, 4 and 5 in Hanks et al. (2004) [22] report showed also PCD, as well as in 6 of those 15 reported cases update [5]. Considering that subjacent genetic cause was demonstrated to MVA, this opens the possibility that *BUB1B *defects can be involved in PCD origin. Mitotic spindle checkpoint serves as a surveillance mechanism that ensures the faithful transmission of chromosomes from a mother cell to its two daughter cells during mitosis [23], this can be involved also in PCD origin. On the other hand, because at least 6 genes have been involved with such checkpoint [23], the origin of PCD may be heterogeneous and should involve a mechanism that triggers the whole set chromosome segregation at mitotic spindle checkpoint.

Other aspect to be considered in PCD origin is the centromere. A basic defect of centromeric region in PCD was suggested [19]. Two essential DNA sequences of the centromere were evaluated by FISH in this family: alpha satellite and CENPB-Box sequences. Centromere function requires the presence of alpha satellite DNA in all human centromeres [24]. We observed alpha satellite DNA from all centromeres and the centromere of chromosomes 1, 13/21, 18, and X, as well as centromeric 17 bp CENPB-Box sequences in prophasic, metaphasic and anaphasic cells from the propositus and his parents. CENPB-Box sequence interacts with the kinetochore protein CENP-B required for the pairing of sister chromatids as structural support and in the conformation of primary constriction and kinetochore [25]. Cytological evidence support the presence of functional centromeres in PCD cells, by positive Cd stain in the propositus and his parents and in one previous PCD report [8]. Also, primary constrictions are evident in this and previous PCD reports. It seems that essential centromere integrity is present and remains unclear if whether or not is involved in PCD origin. Other mechanisms related to cell cycle regulation and functional components of the centromeric region such as defective centromeric cohesion [26], or kinetochore defective proteins are probable.

The PCD trait as a sole chromosome abnormality occurs in healthy individuals. Some authors suggest this PCD trait to be harmless [1,8,18]. In this report four individuals presented PCD, and three of them were phenotypically normal. Noteworthy, all the 30 PCD individuals from 9 previous families included in Table 3 were also phenotypically normal. Individuals with this category of PCD have no a recognizable clinical pattern [6-10]. In three of such families clinical findings were informed and reported as coincidental [6,8,10]. The abnormal phenotype observed in the propositus shows no concordance to previous PCD without MVA cases. PCD trait observed in healthy paternal relatives and all previous cases, represent the common one dose effect of this autosomal dominant trait (OMIM, *176430) [27]. Such mode of inheritance was concordant with this report because male to male transmission was observed. In autosomal dominant PCD and abnormal phenotype associated to MVA, homozygosity was implicated [28,29]. This statement was confirmed by Plaja et al. (2001) [3] in three patients compared with 8 previous cases exhibiting microcephaly, CNS anomalies, mental retardation, prenatal and postnatal growth retardation and cancer, proposing that in vivo occurrence of random aneuploidies and chromosome or genome instability disorder explained some of the clinical data. It seems that variegated aneuploidy is associated to an abnormal phenotype. Our propositus showed prenatal and postnatal growth retardation, profound developmental delay, hypoplasia of the brain and clonic seizures, coincident with MVA reports [5], but our case did not show MVA nor apparent cancer risk. Alternatively, considering that the inheritance of MVA is recessive [5], and some heterozygotes show levels of PCD without variegated aneuploidy, this can be compatible with those individuals described in present report or in previous reports regarding apparently harmless PCD. However, the relationship between PCD and MVA is uncertain [5]. Also, we considered the possibility that neurogical affectation in the patient studied by us could be associated with neonatal hypoxia. In these cases, genetic heterogeneity may be involved.

Association of this PCD trait with abortions and infertility has been reported [6,7,9,10]. This was observed in 12 of 34 PCD individuals from 8 of 10 previous families including present report (Table 3). We observed infertility in two paternal aunts (II-2 and II-3 in fig. 1); cytogenetical analysis was available only in one of them observing PCD. The estimated abortion frequency in descendents of reported PCD individuals was 37% (22 of 60) which is higher than those observed in general population of 15% [30]. In one report, unexplained recurrent abortion observed in both parents with PCD was considered the consequence of abnormal behavior of the centromeres involving probable homozygous effect [9]. Previous observations are coincidental but remark the occurrence of subfertility in PCD individuals.

## Conclusion

Present report represents a new family with PCD as a sole chromosome abnormality. Cell cycle studies revealed that cell cycle reduction could be considered a distinctive finding in these cases. Based in previous reports and the fact that cells of PCD patients were unresponsive to colchicine is probable that a defective colcemid-induced mitotic-spindle checkpoint is involved. Is open the possibility that *BUB1B *defects or other genes involved in such checkpoint may be involved in PCD origin. In this cases considered DNA centromeric sequences were present. It seems that essential centromere integrity is present and remains unclear if whether or not is involved in PCD origin. Other mechanisms related to cell cycle regulation and functional components of the centromeric region may be involved. Interestingly, the PCD trait as a sole chromosome abnormality occurs in healthy individuals and there is not a characteristic associated abnormal phenotype. Only subfertility seems to be a common finding in these families. Those families deserve further investigation in order to understand possible mechanism of this mitotic trait.

## Competing interests

The author(s) declare that they have no competing interests.

## Authors' contributions

AR conceived of the study, and participated in its design and coordination, carried out FISH studies, participated in cytogenetic studies, chromosome analysis, statistical analysis and drafted the manuscript. FS participated in the design and coordination of the study and helped to draft the manuscript. LB participated in the design of the study, helped in clinical activities, helped to draft the manuscript and participated in the statistical analysis. JR participated in clinical activities and drafted the manuscript. CP participated in cytogenetic studies, chromosome analysis and statistical analysis. TG participated in cytogenetic studies, chromosome analysis and statistical analysis. EC participated in the design and coordination of the study, carried out clinical activities and drafted the manuscript. All authors read and approved the final manuscript.

## Pre-publication history

The pre-publication history for this paper can be accessed here:



## Figures and Tables

**Table 1:** Percentages of PCD in repetitive colchicine-treated cultures from family members.

Family member	No. of cultures	Total mitoses scored	Percent PCD
Propositus (III-14)	3	399	10.8
Father (II-11)	3	492	22.35
Mother (II-12)	3	1142	0.175
Paternal aunt (II-3)	2	258	7.0
Paternal aunt (II-8)	8	586	8.36
Paternal aunt (II-9)	9	738	2.9
Paternal aunt (II-10)	7	485	2.47
Maternal aunt (II-14)	7	722	2.7
Maternal aunt (II-15)	7	634	1.73
Controls	10	980	0.87

**Table 2:** Cell cycle durations in paternal sibship with PCD (paternal aunt II-8, propositus III-14, father II-11) versus maternal sibship without PCD (maternal aunts II-14 and II-15, mother II-12).

Groups	Average generation time			
	
	Prophases	Metaphases	Anaphases	Total cells
Paternal sibship with PCD Mean* (SD)**	29.49 (6.54)	29.81 (3.01)	21.79 (0.41)	27.03 (3.05)
Maternal sibship without PCD Mean* (SD)**	24.39 (3.17)	27.97 (1.18)	31.83 (3.95)	27.62 (1.63)
Comparison between groups t value	3.17	1.18	3.95	1.63
Significance	N.S.	N.S.	P < 0.005	N.S.

* = hours, ** = Standard deviation. Statistical test, t-Student for independent samples.

**Table 3:** Main features of published families with PCD and present report.

FEATURES	Rudd et al. 1983 [6]			Gabarrón et al. 1986 [7]	Madan et al. 1987 [8]	Bajnoczky and Gardó 1993 [9]	Keser et al. 1996 [10]			Present report
	A	B	C				A	B	C	
Individuals with PCD	3	4	3	4	4	4	4	3	1	4
Individuals with abortion or infertility	2/3^§§^	0	1/3^§^	2/4^§^	0	2/4^§^	1/4^§^	2/4^§^	1/1^§^	1/4^§§^
PCD in colchicine treated cultures *	14–15	17–62	10–16	5.2–36	6–12	7–38	6–21	6–28	32	5–55
PCD in untreated cultures *	7–20	10–21	6–6.5	8.5–39	0–3	NI	17	7	NI	5
Controls *	0–0.5	0–0.5	0–0.5	1	0–1	4.1–5.2	0–1	0	0	0.87
Cd stain	NI**	NI	NI	NI	+	NI	NI	NI	NI	+
Cell cycle	Short	NI	NI	Short	NI	NI	NI	NI	NI	Short

* = range of percentages. ** = Non investigated. ^§ ^= Individuals with abortion. ^§§ ^= Individuals with infertility.
